# *cifB-*transcript levels largely explain cytoplasmic incompatibility variation across divergent *Wolbachia*

**DOI:** 10.1093/pnasnexus/pgac099

**Published:** 2022-06-28

**Authors:** J Dylan Shropshire, Emily Hamant, William R Conner, Brandon S Cooper

**Affiliations:** Division of Biological Sciences, University of Montana, Missoula, MT 59812, USA; Department of Biological Sciences, Lehigh University, Bethlehem, PA 18015, USA; Division of Biological Sciences, University of Montana, Missoula, MT 59812, USA; Division of Biological Sciences, University of Montana, Missoula, MT 59812, USA; Division of Biological Sciences, University of Montana, Missoula, MT 59812, USA

**Keywords:** Endosymbiosis, host–microbe interactions, reproductive parasitism, *Drosophila*, *Wolbachia*

## Abstract

Divergent hosts often associate with intracellular microbes that influence their fitness. Maternally transmitted *Wolbachia* bacteria are the most common of these endosymbionts, due largely to cytoplasmic incompatibility (CI) that kills uninfected embryos fertilized by *Wolbachia*-infected males. Closely related infections in females rescue CI, providing a relative fitness advantage that drives *Wolbachia* to high frequencies. One prophage-associated gene (*cifA*) governs rescue, and two contribute to CI (*cifA* and *cifB*), but CI strength ranges from very strong to very weak for unknown reasons. Here, we investigate CI-strength variation and its mechanistic underpinnings in a phylogenetic context across 20 million years (MY) of *Wolbachia* evolution in *Drosophila* hosts diverged up to 50 MY. These *Wolbachia* encode diverse Cif proteins (100% to 7.4% pairwise similarity), and AlphaFold structural analyses suggest that CifB sequence similarities do not predict structural similarities. We demonstrate that *cifB*-transcript levels in testes explain CI strength across all but two focal systems. Despite phylogenetic discordance among *cifs* and the bulk of the *Wolbachia* genome, closely related *Wolbachia* tend to cause similar CI strengths and transcribe *cifB* at similar levels. This indicates that other non-*cif* regions of the *Wolbachia* genome modulate *cif*-transcript levels. CI strength also increases with the length of the host’s larval life stage, presumably due to prolonged *cif* action. Our findings reveal that *cifB-*transcript levels largely explain CI strength, while highlighting other covariates. Elucidating CI’s mechanism contributes to our understanding of *Wolbachia* spread in natural systems and to improving the efficacy of CI-based biocontrol of arboviruses and agricultural pests globally.

Significance Statement Host–microbe endosymbioses are the most intimate species interactions, and among all endosymbionts, *Wolbachia* bacteria are the most common. *Wolbachia* prevalence in nature stems largely from its ability to hijack host reproduction to spread. While some *Wolbachia* feminize or kill males, cytoplasmic incompatibility (CI) is the most common manipulation in *Wolbachia's* arsenal. CI strength varies widely among strains, and we report that testes-transcript levels of a single CI gene (*cifB*) largely explains this variation across 20 million years of *Wolbachia* divergence. We also report other factors that contribute to and modulate CI strength. Results reveal predictors of CI-strength variation across divergent *Wolbachia*–host systems, which is crucial to understanding and expanding CI-based biocontrol of arboviruses and agricultural pests on multiple continents.

## Introduction

Endosymbioses are intimate associations where microbes live inside the cells of other organisms (1). This type of interaction led to the evolution of mitochondria and chloroplasts, and thus all eukaryotic life on Earth (2). Maternally transmitted *Wolbachia* are the most common endosymbionts, infecting over half of insect species (3). While some *Wolbachia* are required for host survival (4, 5), many manipulate host reproduction to spread to high frequencies (6–11).

**Fig. 1.**
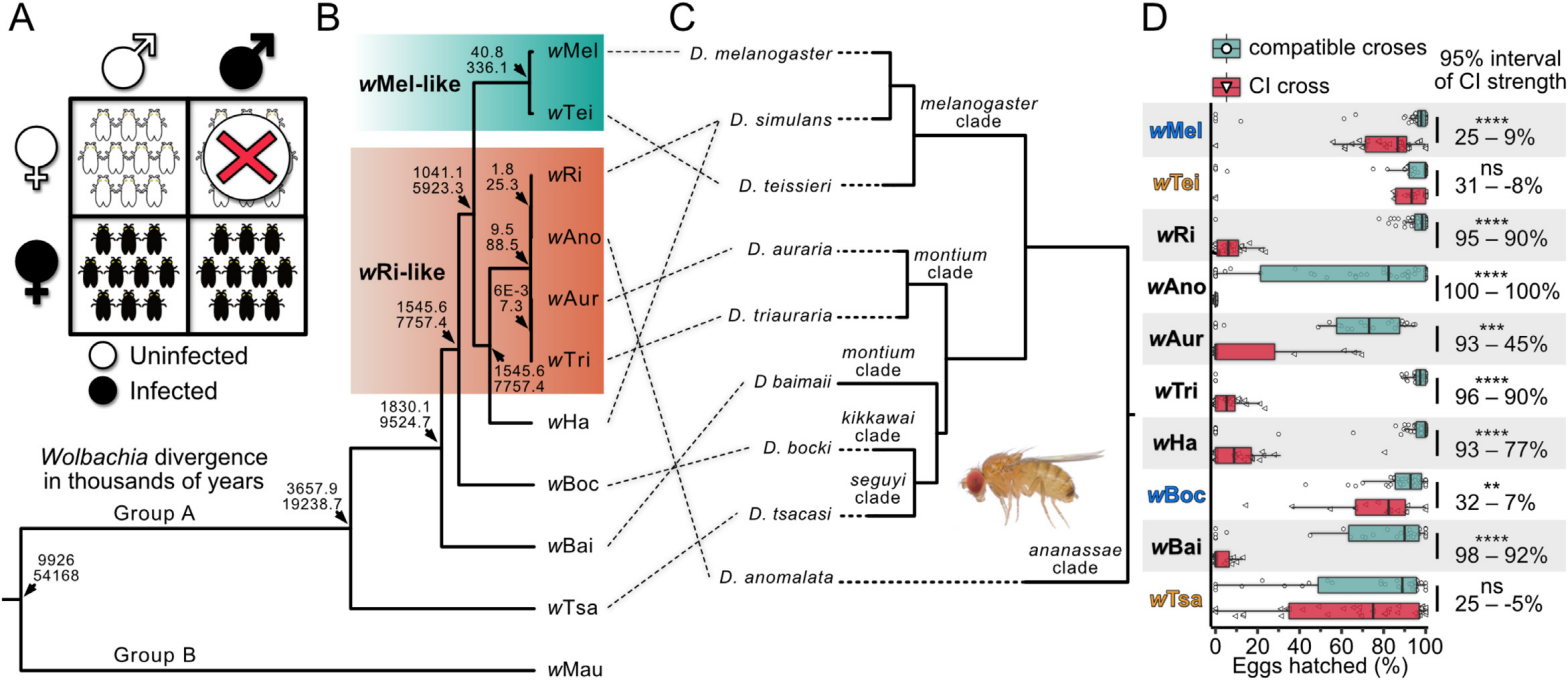
CI phenotype and *Wolbachia* and host phylogenies. (A) CI kills a proportion of eggs when uninfected females mate with infected males. All other crosses are compatible. (B) Bayesian *Wolbachia* chronogram with absolute divergence estimates in thousands of years (95% credible interval of the median) for 10 Group-A *Wolbachia*, with Group-B *w*Mau that infects *D. mauritiana* as an outgroup (37). The chronogram was estimated using 156 full-length and single-copy genes (130,359 bp) of equal length and based on prior calibration of rates of *Wolbachia* divergence (38). All nodes have posterior probabilities >0.95. *w*Mel-like and *w*Ri-like clades are highlighted. (C) Phylogram of *Drosophila* host species used in this study, based on 20 conserved and single-copy genes (38). All nodes have posterior probabilities >0.95. Dashed lines pair *Wolbachia* strains with their *Drosophila* host species and indicate topological discordance. Female *D. teissieri* is displayed to the bottom right. (D) Six *Wolbachia* induced strong CI, two yielded weak CI, and two caused nonsignificant reductions in egg hatch. “Compatible crosses” include the three compatible crosses in Fig. 1A. The *w*Aur compatible cross includes only the infected female x infected male cross since uninfected *w*Aur males were largely inviable. CI strength was calculated as 1—(CI-cross hatch rate/mean hatch of compatible crosses) (10) and displayed as a percentage. 0% CI represents no deviation from average compatibility, and 100% CI represents no eggs hatching. BCa confidence intervals of CI strength are displayed to the right of the plots. Significant differences are based on Mann–Whitney *U* tests between compatible and CI crosses for each strain. Names of strains are displayed in orange text if they do not cause significant CI, blue text if they cause weak CI, and black text if they cause strong CI. Significant differences are *P < 0.05, **P < 0.01, ***P < 0.001, and ****P < 0.0001. Nonsignificant results are denoted with ns. Exact *P*-values are reported in Supplementary Table S1, and Supplementary Fig. S1 displays all cross types individually.

Reproductive manipulations include male-killing, feminization, and parthenogenesis (12). However, cytoplasmic incompatibility (CI) is by far the most common manipulation (Fig. 1A), occurring in at least 10 arthropod orders (13). CI kills uninfected eggs fertilized by *Wolbachia*-infected males. Infected embryos are rescued from CI, providing them a relative fitness advantage that promotes *Wolbachia* spread to high frequencies in host populations (10, 11). Two genes associated with *Wolbachia*’s prophage WO—an integrated temperate bacteriophage in the *Wolbachia* genome—govern CI and rescue: CI factors *A* and *B* (*cifA* and *cifB*). In transgenic expression systems, *cifB* causes CI (14–20), and *cifA* rescues CI (16, 21). In some systems, co-expression of *cifA* and *cifB* in the testes is necessary for CI induction (15, 16, 20). Notably, strong CI directly enables biocontrol programs to transform naturally uninfected mosquito populations with pathogen-blocking *Wolbachia* and reduce population sizes of disease vectors and agricultural pests globally (22–24).

CI strength is highly variable within and among *Wolbachia* strains, ranging from complete to statistically insignificant embryonic lethality for mostly unknown reasons. Within systems, CI strength varies by male age (25, 26), temperature (26), mating history (27), rearing density (28), host genotype (6, 29–31), and other factors. Numerous hypotheses may explain molecular mechanisms of CI-strength variation. Most proximally, high *cifA-* and *cifB-*transcript levels should cause strong CI since more sperm will contain Cif proteins, and more Cifs will reach the fertilized embryo (14–16, 32). However, higher *Wolbachia* density and slower development time should also covary with stronger CI since more *Wolbachia* should produce more Cifs (13), and slower development gives Cifs more time to act or localize (33). Cif protein sequence variation also contributes to CI strength (20, 34, 35). Indeed, theory predicts that CI-causing genes are under weak purifying selection since they are expressed in males that do not transmit *Wolbachia* (36). Weak selection on these genes likely results in high divergence rates and varied enzymatic efficiencies (34, 36).

Despite CI’s importance in *Wolbachia*’s widespread prevalence and use in biocontrol, the evolutionary and mechanistic underpinnings of CI-strength variation remain unresolved. Here, we leverage 20 million years (MY) of *Wolbachia* evolution in *Drosophila* hosts diverged up to 50 MY to assess the extent of CI-strength variation among strains, and its mechanisms, under consistent experimental conditions. We also investigate the sequence and structural variation of proteins that contribute to CI and test whether *Wolbachia* density, *cif*-transcript levels, and/or host developmental time can explain CI-strength variation. While several factors may contribute to CI strength, we report for the first time that *cifB*-transcript levels in testes are the most substantial contributor.

## Results and discussion

### *Wolbachia* phylogeny and host association

Our 10 focal *Wolbachia* include *w*Mel-like variants (*w*Mel of *D. melanogaster* and *w*Tei of *D. teissieri*), *w*Ri-like variants (*w*Ri of *D. simulans, w*Ano of *D. anomalata, w*Aur of *D. Auraria*, and *w*Tri of *D. triauraria*), and several other Group-A strains (*w*Ha of *D. simulans, w*Boc of *D. bocki, w*Bai of *D. baimaii*, and *w*Tsa of *D. tsacasi*) that infect divergent *Drosophila* host species. According to a Bayesian chronogram with absolute age estimates (156 genes; 130,359 bp), Group-A strains diverged from Group-B *Wolbachia* like *w*Mau in *D. mauritiana* 9.9 to 54 MY ago (Fig. 1B), agreeing with past estimates (37). Our 10 Group-A strains encompass 3.6 to 19.2 MY of evolution (Fig. 1B). These *Wolbachia* infect nine *Drosophila* species diverged 10 to 50 MY (Fig. 1C) (38).

### CI strength varies widely among *Wolbachia–Drosophila* associations

Since CI results in embryonic lethality, we measured embryonic hatching and defined CI strength as *s_h_*, where *s_h_* is equal to 1—(CI-cross hatch rate/mean hatch of compatible crosses) (10, 39). Estimates of *s_h_* were made using 3-day-old males that cause relatively strong CI (25, 40). Compatible crosses did not significantly differ in egg hatch for any strain (*P* > 0.05; Supplementary Fig. S1A). Only *w*Tei (95% BCa interval of *s_h_* = −8% to 31%; *P* = 0.11) and *w*Tsa (*s_h_* = −5% to 25%; *P* = 0.55; Fig. 1D) did not cause statistically significant CI. *w*Tei is known to cause very weak CI, although statistically nonsignificant hatch reductions are common and depend on both *Wolbachia* and host variation (6). The remaining eight strains were classified into weak or strong, where strong-CI inducers had less than 20% egg hatch on average. *w*Mel (*s_h_* = 9% to 25%; *P* = 3.5E-09) and *w*Boc (*s_h_* = 7% to 32%; *P* = 2.4E-3; Fig. 1D) caused weak but significant CI, while *w*Ri (*s_h_* = 90% to 95%; *P* = 1.4E-14), *w*Ano (*s_h_* = 100% to 100%; *P* = 1.4E-8), *w*Aur (*s_h_* = 45% to 93%; *P* = 2.2E-4), *w*Tri (*s_h_* = 90% to 96%; *P* = 9.95E-12), *w*Ha (*s_h_* = 77% to 93%; *P* = 1.4E-12), and *w*Bai (*s_h_* = 92% to 98%; *P* = 7.4E-06) caused strong CI (Fig. 1D).

### Cif proteins are highly diverged, and CifB evolves faster than CifA

**Fig. 2.**
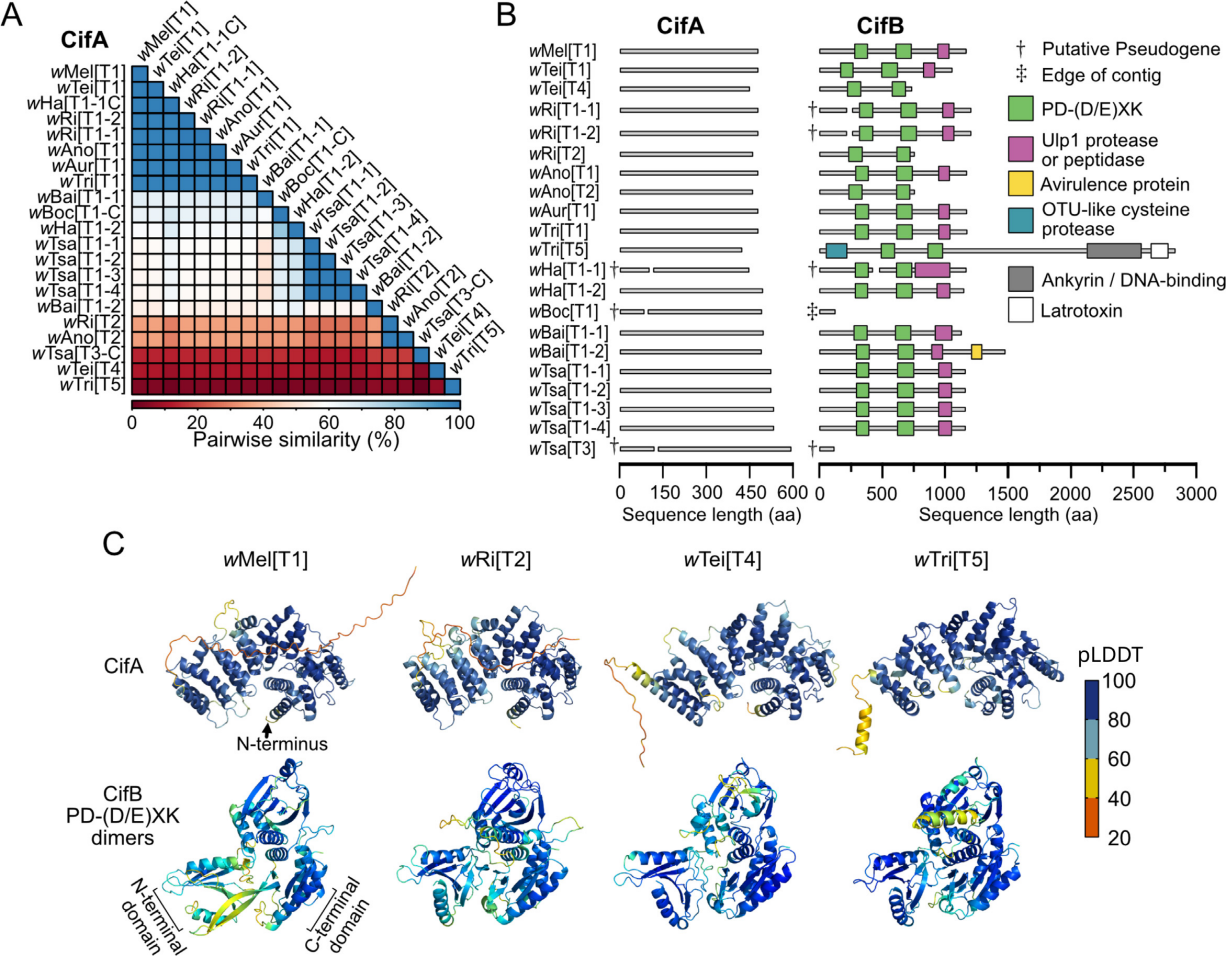
CifA and CifB variation. (A) Similarity matrix displaying pairwise amino acid similarity for CifA proteins ranging from 100% to 15.4%, displaying considerable genetic diversity. (B) CifA (left) and CifB (right) protein architecture with HHpred annotations with *P* > 80% are displayed as large colored boxes. CifA had no confident annotations. The PD-(D/E)XK pair was the only feature represented in all CifB structures. (C) AlphaFold structures of CifA and CifB’s PD-(D/E)XK pair from four divergent Cif Types. Proteins are colored by pLDDT—a metric of structure confidence. pLDDT scores range from 0 to 100 where pLDDT > 90 is high confidence, 90 > pLDDT > 70 is confident, 70 > pLDDT > 50 is low confidence, and pLDDT < 50 is very low confidence.

*cifs* are classified into five phylogenetic clades (Types 1 to 5) (41), each thought to contribute to CI (13, 18, 20). All focal *Wolbachia* genomes contained one to five *cifA* and *cifB* pairs, including at least one pair of Type 1 *cifs* in each genome (*cif_[T1]_*; Supplementary Fig. S1B). Type 2 (*w*Ri and *w*Ano), Type 3 (*w*Tsa), Type 4 (*w*Tei), and Type 5 (*w*Tri) were also represented across strains (Supplementary Fig. S1B). CifA (*N* = 21) and CifB (*N* = 19) protein sequences were aligned, and pairwise identity was calculated for shared sites. CifA protein sequences ranged from 100–15.4% identity (Fig. 2A), where CifA_[T1]_ copies shared between 100% and 60.8% identity with other CifA_[T1]_ proteins and between 45.2% and 18.2% identity with CifA_[T2–T5]_ proteins (Fig. 2A). Conversely, CifB sequences ranged from 100% to 7.4% identity, where CifB_[T1]_ shared between 100% and 29.7% identity with other CifB_[T1]_ proteins and between 26% and 7.4% identity with CifB_[T2–T5]_ proteins (Supplementary Fig. S1C).

We annotated functional domains using HHpred (42). CifA had no hits above 80% probability—our threshold for accepting annotations (Fig. 2B; Supplementary Table S2). The lack of confident annotations for CifA indicates that CifA either has a novel function not currently represented in the database or, more likely, that it is too diverged from database proteins to be confidently annotated. However, CifB had six confident domain predictions (Supplementary Table S2): PD-(D/E)XK nuclease, Ulp1 protease or peptidase, Avirulence protein, OTU-like cysteine protease, Ankyrin/DNA-binding, and Latrotoxin (Fig. 2B). The last three domains were unique to CifB*_w_*_Tri[T5]_, and the avirulence protein domain was unique to CifB*_w_*_Bai[T1–T2]_. The Ulp1 protease domain, known to be a functional deubiquitinase (14), was present in all CifB_[T1]_ proteins and absent in other CifB Types. Deubiquitinase activity does not appear to be essential for CI induction (32). Despite considerable sequence variation (Supplementary Fig. S1C), a pair of PD-(D/E)XK-nuclease domains was present in all CifB proteins—CifB_[T3 and T4]_ are functional nucleases (18, 19), and CifB_[T1]_ is a DNase with unknown catalytic residues (43). We conclude that Cifs are divergent in both sequence and domains but with several shared features. Indeed, the representation of the PD-(D/E)XK pair in all CifB Types suggests it is functionally important (13, 43) for CI and/or another unknown phenotype.

### CifB sequence and structural similarities do not covary

Theory predicts that CI-causing genes like *cifB* are under weak purifying selection since they are expressed in males that do not transmit the infection, while rescue genes like *cifA* should be preserved (36). Indeed, genomic and phenotypic analyses of putatively pseudogenized Cifs suggest that CifB has a higher rate of putative pseudogenization than CifA (37, 41). For example, non-CI-causing *w*Mau has a putatively pseudogenized CifB, intact CifA, and can rescue CI induced by closely related strains (37, 44). Consistent with these results, our CifA sequences (95% CI of the median = 57.5% to 63.9% identity) were significantly more similar than were CifB sequences (95% CI = 41.9% to 55.0%) according to a Wilcoxon matched-pairs signed-rank test (*P* < 0.0001). However, this result may also be explained by relaxed selection due to structural flexibility in the protein.

To test this hypothesis, we generated AlphaFold (45) CifA structures, and CifB structures surrounding the shared PD-(D/E)XK pair (CifB_nuc_), for representative Cif proteins from Types 1, 2, 4, and 5 (Fig. 2C). Type 3 was excluded because Type 3 *w*Tsa was putatively pseudogenized (see below), and only the PD-(D/E)XK-nuclease pair of CifB (CifB_nuc_) was analyzed as it was shared across all Types. AlphaFold confidence, measured as a Local Distance Difference Test (pLDDT) score, suggests confident structural predictions (pLDDT = between 74.9 and 86.9; Supplementary Fig. S2; Supplementary Table S3). However, the C-terminus of all CifA protein predictions were low confidence and structurally disordered in the *w*Mel, *w*Ri, and *w*Tei proteins (Fig. 2C; Supplementary Fig. S2A–D). Low-confidence structures can be evidence of disordered protein regions (45). Indeed, empirically-determined crystal structures of CifA-CifB complexes could not resolve CifA’s C-terminus, indicating it is disordered (46). Notably, disordered regions are often capable of forming structures under particular binding associations and may exhibit numerous context-dependent structures that add function to the protein. The CifA proteins formed a concave structure composed primarily of *α*-helices. In contrast, CifB proteins were composed of *α*-helices and *β*-strands organized into three regions: N-terminal PD-(D/E)XK, C-terminal PD-(D/E)XK, and a linker region (Fig. 2C). The *αβββαβ* structural motif typical of PD-(D/E)XK domains (47) was present in both domains in all of the CifB_nuc_ structures.

We generated pairwise alignments of protein structures and calculated template modeling (TM) scores representing structural similarity (48). CifA TM scores positively covaried with percent sequence identity according to a Pearson correlation (*R^2^* = 0.94; *P* = 0.005; *N* = 6 comparisons). However, CifB_nuc_ TM scores did not covary with sequence identity (*R^2^* = 0.17; *P* = 0.74; *N* = 6). These results suggest that CifA protein sequence similarity is a good indicator of structural similarity, whereas CifB_nuc_ sequence similarity is not a good predictor of structural similarity. This is consistent with findings that PD-(D/E)XK domains are structurally conserved but represented by highly divergent sequences (47). These results indicate that CifB’s relatively rapid sequence evolution is likely due to the sequence flexibility underlying CifB’s structure. Since theory predicts that selection will not maintain the CI-causing genes, it is unknown why CifB’s nuclease is structurally maintained. However, it is plausible that the selective maintenance of these structures is dependent on their involvement in other cellular phenotypes such as autophagy (49).

### Cif pseudogenization does not explain non-CI-inducing strains

Two of our *Wolbachia* (*w*Tei and *w*Tsa) failed to cause statistically significant embryonic mortality in the CI crosses. We tested the hypothesis that non-CI inducers had pseudogenized Cif proteins (37, 41) by searching for premature stop codons that truncate the proteins (Fig. 2B). While *w*Tei appeared to have normal Cif proteins, *w*Ri, *w*Ha, *w*Boc, and *w*Tsa encoded at least one Cif copy that was putatively pseudogenized. Three CifA copies are truncated (*w*Ha[T1], *w*Boc[T1], and *w*Tsa[T3]), and four CifB copies are truncated (*w*Ri[T1–1], *w*Ri[T1–2], *w*Ha[T1], and *w*Tsa[T3]). Alternative upstream start codons exist for all but the pseudogenized CifB*_w_*_Tsa[T3]_, which has a transposon inserted after the 348th nucleotide. We conclude that statistically nonsignificant *w*Tei and *w*Tsa CI cannot be explained by pseudogenization alone since *w*Tei’s Cifs appear normal and *w*Tsa has four putatively intact Cif_[T1]_ pairs. Prior genomic and functional analyses of *w*Yak-clade *Wolbachia* support our *w*Tei result (6, 34). However, sequence variation may still contribute to inefficiencies in protein function. Indeed, transgenic expression and biochemical assays revealed that a single Valine to Leucine substitution in CifB*_w_*_Yak[T1]_, a very close homolog of CifB*_w_*_Tei[T1]_, significantly inhibits enzymatic activity relative to sister *w*Mel (34). Finally, *w*Boc was the only strain that lacked an intact Cif pair. Two hypotheses could explain why *w*Boc induced weak CI despite pseudogenized Cifs. First, expression of the upstream coding sequence may be sufficient to yield a phenotype. Second, since complete circularized genomes were only available for *w*Mel, *w*Ri, and *w*Ha, it is plausible that the *w*Boc genome contains additional unknown *cif* copies that our analyses did not detect.

### *Wolbachia* densities in testes do not fully explain CI strength

**Fig. 3.**
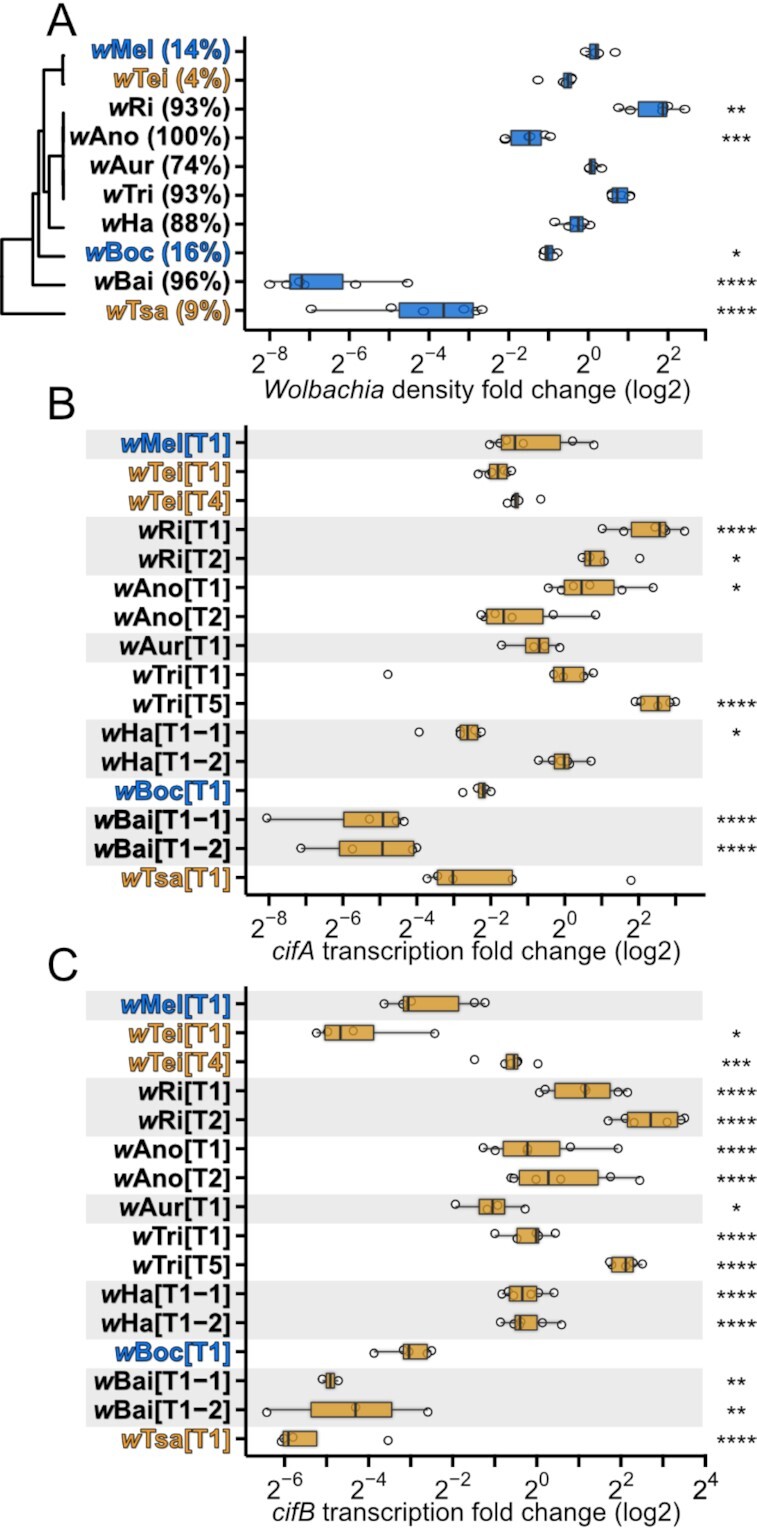
*Wolbachia* densities and *cif*-transcript levels in testes vary for *Drosophila*-associated *Wolbachia*. (A) *Wolbachia*-density fold change (FC) from testes DNA extracts was calculated as 2^–∆∆CT^ of the *Wolbachia* gene *ftsZ* to the host gene *mid1* relative to a randomly selected *w*Mel-infected sample. Notably, *w*Ano and *w*Bai are strong-CI *Wolbachia* that have significantly lower *Wolbachia* densities than weak-CI *w*Mel. *Wolbachia* relationships presented in Fig. 1B and mean BCa CI-strength estimates in parentheses are displayed for reference. (B) *cifA-* and (C) *cifB*-transcript levels in testes of infected flies of each strain. *cif*-transcript level FC was calculated as 2^–∆∆CT^ of *cif*-transcript levels to an exogenous spike-in control relative to the average transcript levels of *cif_wMel[T1]_, cif_wRi[T2]_, cif_wTei[T4]_*, and *cif_wTri[T5]_* samples. Names of strains are displayed in orange text if they do not cause significant CI, blue text if they cause weak CI and black text if they cause strong CI. Significant differences are based on FDR-adjusted pairwise t tests relative to *w*Mel abundance in A and *cif_wMel[T1]_* transcript levels in B and C. *P < 0.05, **P < 0.01, ***P < 0.001, and ****P < 0.0001. Exact *P*-values are reported in Supplementary Table S1.

The bacterial density hypothesis predicts that CI strength covaries with *Wolbachia* densities in testes (13), though there are exceptions where strong-CI-inducing strains have unusually low densities (50). We tested the bacterial density hypothesis by measuring *Wolbachia* densities in whole-testes extracts via qPCR (Fig. 3A) and performing categorical and qualitative correlation analyses with CI strength.

First, we predicted that strong-, weak-, and non-CI-inducing *Wolbachia* would have higher, similar, and lower *Wolbachia* densities in testes than model *w*Mel that caused weak CI. However, only one strong-CI-inducing strain, *w*Ri, occurred at statistically higher densities in testes than *w*Mel (Fig. 3A). Three of the other strong-CI-inducing strains (*w*Aur, *w*Tri, and *w*Ha) had similar densities, and two had lower densities (*w*Bai and *w*Ano) compared to *w*Mel-infected testes. Contrary to our prediction, the other weak-CI line, *w*Boc, also occurred at lower densities in testes than *w*Mel. Finally, non-CI-causing *w*Tei and *w*Tsa had similar and lower densities than *w*Mel, respectively (Fig. 3A). Intriguingly, despite diverging from *w*Ri on the order of thousands of years ago (Fig. 1B), strong-CI-inducing *w*Ano and *w*Aur occur at lower densities in their hosts, relative to *w*Ri in *D. simulans*. In summary, only *w*Ri and *w*Tsa fit our predictions for categorical assignment (Table 1), and low-density *w*Ano, *w*Boc, and *w*Bai caused stronger CI than predicted by their testes *Wolbachia* densities.

**Table 1.** Summary of CI-strength, *Wolbachia*-density, and *cif*-transcript level results.

***Wolbachia*of host**	**CI strength**	*Wolbachia* **density**	***cifA* level**	***cifB* level**
*w*Mel of *D. melanogaster*	Weak	**Medium**	**Medium**	**Medium**
*w*Tei of *D. teissieri*	None	Medium	Medium/Medium	Low/High
*w*Ri of *D. simulans*	Strong	**High**	**High/High**	**High/High**
*w*Ano of *D. anomalata*	Strong	Low	**High/Medium**	**High/High**
*w*Aur of *D. auraria*	Strong	Medium	Medium	**High**
*w*Tri of *D. triauraria*	Strong	Medium	**High/Medium**	**High/High**
*w*Ha of *D. simulans*	Strong	Medium	Low/Medium*	**High/High**
*w*Boc of *D. bocki*	Weak	Low	**Medium**	**Medium**
*w*Bai of *D. baimaii*	Strong	Low	Low/Low	Low/Low
*w*Tsa of *D. tsacasi*	None	**Low**	Medium	**Low**

*Note:* High, medium, and low density/*cif* transcription is defined as higher, similar, and lower than the weak-CI-causing *w*Mel. We predicted that *Wolbachia*density/*cif* transcription would be higher, similar, and lower than *w*Mel for strong, weak, and non-CI inducers. Measures consistent with these predictions are bolded.

*Co-expression of *w*Ha's two *cifA* copies may yield higher total expression than *cifA_wMel[T1]_*, but neither copy alone had higher transcript levels.

We next tested the bacterial density hypothesis using a phylogenetic generalized least squares (PGLS) regression that accounts for phylogenetic signal and dependencies within the residuals of our comparisons. *Wolbachia* densities in testes had a statistically nonsignificant relationship with CI strength among our focal *Wolbachia* (*R^2^* = 0.03; *P* = 0.62; Supplementary Table S4). However, we also subset our data by removing non-CI inducers (*w*Tei and *w*Tsa) and/or low-density strains (*w*Ano, *w*Boc, *w*Bai, and *w*Tsa), since they seemingly yielded stronger CI than expected from their densities. Removal of low-density *Wolbachia* revealed a strong positive relationship between *Wolbachia* densities and CI strength (*R^2^* = 0.99; *P* = 2.56E−05; Supplementary Table S4). Similarly, removal of non-CI strains recovered a strong correlation between densities and CI strength (*R^2^* = 0.99; *P* = 9.74E−07; Supplementary Table S4). We conclude that *Wolbachia* densities in the testes cannot fully explain CI-strength variation among our ten systems, but it trends with CI strength among a subset of strains. Notably, while *Wolbachia* densities measured via qPCR from whole-testes extracts is a poor predictor of CI strength across systems, it remains plausible that *Wolbachia* density in specific stages of spermatogenesis is a strong predictor (51).

### *cifB*-transcript levels in testes largely explain CI strength

The *Wolbachia*-density hypothesis is based on the presumption that CI-gene expression positively covaries with *Wolbachia* abundance in host tissues (13). Thus, we tested if *cifA-* and/or *cifB*-transcript levels in whole-testes extracts covaried with CI strength using RT-qPCR. As with *Wolbachia* densities, we tested if *cif*-transcript levels and CI strength covaried via categorical and/or qualitative analyses. Relative to weak-CI-causing *w*Mel, we predicted that strong-, weak-, and non-CI strains would have higher, similar, or lower *cif*-transcript levels, respectively.

Regarding *cifB*-transcript levels, seven of nine strains matched our predicted transcript levels in testes relative to *cifB_wMel[T1]_*: Five strong-CI strains (*w*Ri, *w*Ano, *w*Aur, *w*Tri, and *w*Ha) had copies with only higher transcript levels, the weak-CI *w*Boc had similar transcript levels, and the non-CI-inducing *w*Tsa had significantly lower transcript levels (Fig. 3C). Of the remaining two strains, strong-CI *w*Bai only had copies with significantly lower transcript levels, and *w*Tei that did not cause statistically significant CI had a copy with lower transcript levels (*cifB_wTei[T1]_*) and one with higher transcript levels (*cifB_wTei[T4]_*; Fig. 3C). Finally, PGLS regression revealed a significant positive relationship between *cifB_[T1]_*-transcript levels and CI strength when low-density *Wolbachia* (*R^2^* = 0.33; *P* = 0.04) and non-CI-inducing strains were removed from the analysis (*R^2^* = 0.89; *P* = 4.66E−03; Supplementary Table S4). A single *cifB* copy (*cifB_[T1]_*) was selected for analysis from each strain since Type 1 is the only *cifB* Type maintained across all strains.

Conversely, only five strains matched our predicted transcript levels pattern relative to *cifA_wMel[T1]_* in testes: Three strong-CI strains (*w*Ri, *w*Ano, and *w*Tri) had at least one *cifA* copy with higher transcript levels, the weak-CI strain *w*Boc had similar transcript levels, and the strong-CI strain *w*Ha had copies with similar and lower transcript levels that likely amount to higher combined transcript levels (Fig. 3B). The remaining five strains had higher (*w*Tei and *w*Tsa) and lower (*w*Ano and *w*Bai) transcript levels than predicted based on their CI strength (Fig. 3B). A PGLS regression revealed no significant relationship between *cifA_[T1]_*-transcript levels and CI strength (Supplementary Table S4). In summary, categorical analysis of *cifA*-transcript levels relative to *w*Mel suggests that CI strength can be explained by *cifA*-transcript levels in the testes of *w*Ri, *w*Ano, *w*Tri, *w*Boc, and *w*Ha but not *w*Tei, *w*Ano, wBai, and *w*Tsa (Table 1). The quantitative analysis supports the disconnect between *cifA_[T1]_*-transcript levels and CI strength but does not account for the combinatory impact of expressing multiple *cifA* copies. We also tested for a relationship between *cif*-copy number and CI strength and found no significant relationship (*R^2^* = 0.11; *P* = 0.76).

In sum, our analyses reveal for the first time that *cifB*-transcript levels in testes largely explains CI-strength variation. However, two strains are interesting exceptions: *w*Tei did not cause statistically significant CI, yet *cifB_wTei[T4]_*-transcript levels were relatively high, and *w*Bai caused very strong CI, but *cifB_wBai[T1–1]_*- and *cifB_wBai[T1–2]_*-transcript levels were much lower than expected. We propose four non-exclusive hypotheses for these patterns. First, we measured transcript levels as a proxy for translation, but the two can be decoupled (52). Thus, *w*Tei may produce fewer proteins per transcript than other strains, and *w*Bai may produce more proteins per transcript. Second, we measured *Wolbachia* densities and *cif*-transcript levels from full-testes extracts, but *Wolbachia* localization within spermatogenesis is linked to CI-strength variation (51). It is plausible that *w*Tei is poorly localized for CI induction while *w*Bai is optimally localized. Third, theory and empirical studies agree that hosts develop resistance to CI (6, 36, 40). Since most of the strains investigated were from different species, it is plausible—indeed, perhaps likely—that host factors modulate CI-strength variation. Future work aimed at determining the contributions of host factors to CI strength and the relationship between *cifB*-transcript levels and CI strength will be particularly useful. Finally, *cif*-genetic diversity may contribute to variation in CI efficiencies. This hypothesis seems particularly likely for *w*Tei since a single amino acid substitution between CifB*_w_*_Mel[T1]_ and a CifB*_w_*_Tei[T1]_ homolog in *w*Yak inhibits transgenic enzymatic activity and CI penetrance (34). CifB*_w_*_Bai[T1–2]_ uniquely encodes a domain with structural homology to the cysteine protease avirulence protein AvrPphB of *Pseudomonas* (Fig. 2B). AvrPphB cleaves host kinases and inhibits *Arabidopsis* defenses against *Pseudomonas* colonization (53). Thus, the CifB*_w_*_Bai[T1–2]_ avirulence domain may interfere with host CI-resistance mechanisms (54). However, the molecular function of the AvrPphB-like residues retained in CifB*_w_*_Bai[T1–2]_ and the means of CI suppression are unknown. Future work will be necessary to determine the relative contributions of translation, localization, and genetic variation to CI caused by these extraordinary strains.

### Strong CI and *cifB_[T1]_*-transcript levels show evidence of *Wolbachia* phylogenetic signal

We tested whether closely related *Wolbachia* exhibit similar CI strengths and/or *Wolbachia* densities/*cif*-transcript levels in testes. We first calculated Fritz and Purvis’ *D* statistic (55), which assesses phylogenetic signals of binary traits. *D* = 1 represents a randomly distributed trait across the phylogeny, while *D* = 0 suggests the trait is clumped as if evolving under Brownian motion (i.e. phylogenetic signal). *P*_1_ and *P*_0_ indicate the probability that the *D* value differs from *D* = 1 and *D* = 0, respectively. Causing strong CI (*D* = −0.70; *P*_1_ = 0.01; *P*_0_ = 0.78; Supplementary Fig. S3A) and high*-cifB_[T1]_* transcription (*D* = −0.26; *P*_1_ = 0.02; *P*_0_ = 0.62; Supplementary Fig. S3B) showed evidence of phylogenetic clumping. Conversely, causing CI, having low-*Wolbachia* densities, or expressing high levels of a *cifA_[T1]_* copy was randomly distributed across the phylogeny (Supplementary Fig. S3C–E). However, Geiger simulations suggest that the inclusion of more *Wolbachia*-infected systems may help differentiate our *D* values for low-*Wolbachia* density, *cifA_[T1]_*-transcript levels, and CI induction from randomness (Supplementary Fig. S3), although the sample sizes required are notably unreasonable. For non-proportional data (i.e. CI-strength measures), we also calculated the maximum-likelihood value of Pagel’s lambda (*λ*) (56), using the continuous measures of our traits (*λ* = 0 indicates no phylogenetic signal). This approach supported the finding that *cifB_[T1]_*-transcript levels have phylogenetic signal (*λ* = 0.88; *P* = 0.03), and that *cifA_[T1]_*-transcript levels (*λ* = 0.68; *P* = 0.73), and *Wolbachia* density (*λ* = 0.84; *P* = 0.12) do not. In summary, these results suggest that *Wolbachia* that cause strong CI and have high *cifB*_[T1]_ transcription tend to be closely related. We hypothesize that non-*cif* regions of the *Wolbachia* genome modulate *cifB*-transcript levels. Future work is required to determine if this is via active modulation of *cifB* expression or the indirect effect of loci that regulate *Wolbachia* abundance in host tissues.

### Larval, but not pupal, development time positively covaries with CI strength

*Cardinium*-induced CI strength (33) positively covaries with the length of *Encarsia* wasp pupation time. Conversely, longer development time covaries with weaker CI in *w*Mel-infected *D. melanogaster* (28). Here, we test for the first time whether *Wolbachia*-induced CI strength across numerous *Wolbachia*-*Drosophila* systems covaries with larval (Supplementary Fig. S4A), pupal (Supplementary Fig. S4B), and/or egg-to-adult development times (Supplementary Fig. S4C). A PGLS regression revealed a significant positive relationship between CI strength and the length of the larval life stage (*R^2^* = 0.47; *P* = 0.041), but not with the length of pupation (*R^2^* = 0; *P* = 0.98) or with overall egg-to-adult development time (*R^2^* = 0.21, *P* = 0.22; Supplementary Table S5; Supplementary Fig. S4D–F). Removing low-density *Wolbachia* strains resulted in an even stronger relationship between larval development and CI strength (*R^2^* = 0.86, *P* = 0.02) and revealed a weak yet statistically significant relationship with egg-to-adult development time (*R^2^* = 0.78, *P* = 0.048; Supplementary Table S5; Supplementary Fig. S4G–I), likely driven by the larval pattern. In summary, we find that larval development time is a predictor of *Wolbachia*-induced CI across systems. We present two hypotheses for this correlation. First, as has been proposed for *Cardinium* (57, 58), longer development may enable CI products to accumulate or provide more time to act on their host targets in specific life stages. Second, development time may covary with other contributors of CI-strength variation, including the accessibility of host targets. Future investigations of the developmental restrictions of CI will be necessary to determine the mechanistic basis of the relationship between host development time and CI strength. Moreover, while these data support a relationship between development time and CI strength across systems, it remains to be determined if slow and fast developing males from the same *Wolbachia–Drosophila* system cause different CI strengths.

## Conclusions

By leveraging CI diversity across 20 MY of *Wolbachia* and 50 MY of host evolution, we discovered that *cifB*-transcript levels largely explain CI-strength variation. This result confirms long-held predictions and enables future in-depth mechanistic investigation of the dynamic relationships between transcript levels, density, and localization on CI strength. Our analyses reveal five additional impactful findings. First, our focal *Wolbachia* encode considerable Cif-sequence variation, with some strains having as little as 7.4% pairwise similarity. Second, CifB sequence evolves faster than CifA, but the similarity in the protein structures of CifB nuclease pairs does not covary with CifB sequence similarities. Third, *Wolbachia* densities and *cifA*-transcript levels in testes fail to explain CI strength broadly. Fourth, CI tends to be stronger when larval development time is longer. Finally, *cifB* and strong-CI expression are similar among closely related *Wolbachia*, suggesting that other regions of the *Wolbachia* genome modulate *cif*-transcript levels, either actively or indirectly via effects on *Wolbachia* abundance in host tissues. This latter finding is particularly notable given that *cifs* are associated with prophage-WO regions that tend to be phylogenetically discordant from the bulk of the *Wolbachia* genome. Taken together, these results inform the mechanism and evolution of CI, bringing us closer to developing a complete understanding of CI-strength variation and how it governs *Wolbachia*’s standing as the world’s most common endosymbiont.

## Materials and methods

### *Wolbachia* chronogram

Genomes used are listed in Supplementary Table S6 (6, 37, 38, 59–61). We used Prokka v. 1.11 to annotate genomes, then extracted all single-copy genes that were the same length in each genome for phylogenetic analyses. 156 genes met these criteria. The genes were aligned with MAFFT v. 7 (62), then concatenated. RevBayes v. 1.1.1 was used for all phylogenetic analyses (63). We generated a *Wolbachia* absolute chronogram using the same GTR+Γ model as (38), except that we used a relaxed clock with a branch rate prior of Γ(7,7) for each branch, normalized to a mean of 1 across all branches. Briefly, we assumed a constant-rate sampled-birth-death process, which has four parameters: speciation rate, extinction rate, sampling probability, and the age of the root. We used previously reported speciation and extinction rate estimates based on empirical information (38). Specifically, we previously specified empirical lognormal hyperpriors to determine the means of these distributions so that the prior expected number of species under the birth–death process was equal to the known number of species in the group. The sampling fraction, *ρ*, was set to 0.1. To estimate absolute node ages, we used prior estimates of substitutions per site per year (64). We refer readers to (38) for a detailed description of these methods.

### Host phylogram

We constructed a host phylogram with 20 nuclear genes (a*conitase, aldolase,bicoid,ebony,enolase,esc,g6pdh,glyp,glys,ninaE,pepck,pgi,pgm,pic,ptc,tpi,transaldolase,white,wingless*, and*yellow)*. Coding sequences for these genes in *D. melanogaster* and *D. simulans* were obtained from FlyBase. Orthologs in *D. teissieri* (65), *D. auraria* and *D. triauraria* (66), *D. baimaii, D. bocki*, and *D. tsacasi* (67), and *D. anomalata* (38) were obtained with BLAST using the *D. melanogaster* sequences. The resulting sequences were aligned with MAFFT v. 7 (62) and hand curated to remove fragments of introns that could cause frameshifts.

We used these genes to estimate a phylogram using RevBayes v. 1.1.1, following (38). We used a GTR+Γ model with four rate categories, partitioning by gene and codon position. Each partition had an independent rate multiplier with prior Γ(1,1) [i.e. Exp(1)], as well as stationary frequencies and exchangeability rates drawn from flat, symmetrical Dirichlet distributions [i.e. Dirichlet(1,1,1. . .)]. The model used a uniform prior over all possible topologies. Branch lengths were drawn from a flat, symmetrical Dirichlet distribution and thus summed to 1. Since the expected number of substitutions along a branch equals the branch length times the rate multiplier, the expected number of substitutions across the entire tree for a partition is equal to the partition’s rate multiplier. Four independent runs were performed, and all converged to the same topology. For additional details on the priors and their justifications, consult (38).

### Fly lines, care, and maintenance

Fly lines used are listed in Supplementary Table S6. Uninfected flies were derived via tetracycline treatment in prior studies (15, 68). Tetracycline cleared lines were used in experiments over a year after treatment, avoiding the effects of antibiotic treatment on mitochondria metabolism and density (69). Infection status was confirmed with PCR of the *Wolbachia* surface protein gene. An arthropod-specific 28S rDNA was amplified as a control for DNA quality (6, 37). DNA was extracted for symbiont checks using a squish buffer protocol, described in (25). Flies were reared in vials with 10 mL of food made of cornmeal, dry corn syrup, malt extract, inactive yeast, soy flour, and agar (25). Fly stocks were maintained at 23°C before and during experiments. Flies were anesthetized using CO_2_ for virgin collections and dissections. During hatch-rate assays, flies were mouth aspirated between vials.

### Hatch-rate and egg-lay assays

Four crosses were performed for each strain (Fig. 1A). The one exception was *w*Aur-infected *D. Auraria*, where only crosses with infected males were performed as uninfected males were sickly and failed to mate in pairs. Virgin 6–8-d-old females were added to vials containing a spoon filled with fly food—one fly was added per vial. Food for these assays was the same as standard rearing media but with blue food coloring and 1% extra agar. Fresh yeast paste (3:2 water to yeast) was smeared on the food. After 4–5 h of acclimation, a single 3-d-old virgin male was added to each vial and then incubated overnight. In the morning, flies were aspirated into new vials with a fresh spoon. Vials were incubated for another 24 h before flies were removed via aspirating. Eggs were counted on spoons immediately after flies were removed, and the remaining unhatched eggs were counted after 48 h. The hatch rate was calculated as the proportion of eggs hatched per spoon in 48 h. CI strength (*s_h_*) was measured for each sample as 1—(CI-cross hatch rate/mean hatch of compatible crosses) (10). 95% BCa confidence intervals and means of CI strength were calculated using boot in R (70). We used Mann–Whitney *U* tests in R to determine if hatching differed between CI and compatible crosses for each strain. A Kruskal–Wallis followed by Dunn’s test was performed to determine if any cross types differed. Samples with fewer than ten eggs laid were excluded from hatch-rate analyses.

CI strength is amenable to variation in male age (25, 40), rearing density (28), paternal grandmother age (71), and temperature (72). To enable comparison among different *Wolbachia*–host associations, we controlled these factors. Virgin males were aged for 3 d before pairing with females since younger *D. tscacasi* and *D. bocki* males failed to mate and lay. Flies were given only ∼24 h to lay in vials that would yield fathers to prevent overcrowding. All flies were maintained at 23°C before the experiment, as virgins, and during the experiment. Finally, while we did not control paternal grandmother age, all males in these experiments were derived from females that were not maintained as virgins. Since the paternal grandmother age effect is significantly inhibited after mating, we predict that variation in non-virgin grandmother age has no impact on these studies.

### Cif sequence and structure analyses

*cif* sequences were retrieved from the genomes of all strains used in this study with BlastP. Representative *cifA* and *cifB* genes from each of the five phylogenetic Types of *cif* genes were used as query sequences, based on previous assignments (41): *cifA_w__Mel[T1]_, cifB_w__Mel[T1]_, cifA_w__Ri[T2]_, cifB_w__Ri[T2]_, cifA_w__No[T3]_, cifB_w__No[T3]_, cifA_w__Pip[T4]_, cifB_w__Pip[T4]_, cifA_w__Tri[T5]_*, and *cifB_w__Tri[T5]_. cifs* were assigned a Type designation based on their closest hit from the query sequences and the Type of their nearby *cifA* or *cifB* partner. Glimmer 3 was used to identify the coding region surrounding each blast hit in Geneious Prime and then translated (73).

Three methods were used to characterize Cif proteins further. First, MUSCLE alignments for CifA and CifB were generated in Geneious Prime (73) and were used to create heatmaps of pairwise protein similarity in R using the corrplot package (74, 75). Second, the HHpred webserver (42) was used to identify protein domains. Databases used were SCOPe70_2.07, Pfam-A_v33.1, COG_KOG_v1.0, and SMART_v6.0. Annotations with *P* > 80 were recorded. If multiple hits were retrieved for a single region, only the highest probability annotation was taken and used for annotation.

Finally, ColabFold (76), an AlphaFold2 (45) Google Collaboratory notebook, was used to generate tertiary structures for CifA and CifB’s PD-(D/E)XK domains. Full CifB proteins were not generated due to restrictions on protein length and computational power. Tertiary structure similarity was determined using the Zhang-lab TM-score webserver (48). Each pairwise comparison was conducted twice—switching the order of template and experimental structures—and the average was taken. The relationship between Cif sequence and structural similarity was assessed using a Wilcoxon matched-pairs signed-rank test in Graphpad Prism 8.

### Tissue collection and nucleotide purification

Siblings from hatch-rate assays were collected for DNA and RNA extractions for *Wolbachia*-density and *cif*-transcript-level assays. All tissue was collected the day after crossing for the hatch-rate experiment. Virgin males were CO_2_ anesthetized, and testes were dissected in chilled phosphate-buffered saline. Five pairs of testes were placed into a single tube for DNA extractions and stored at −80°C until processing. The tissue was homogenized, and DNA was extracted with the DNeasy Blood and Tissue kit (Qiagen). For RNA extractions, 15 pairs of testes were placed into a single tube containing 200 µL of Trizol and four 3 mm glass beads. Samples were then homogenized using a TissueLyser II (Qiagen) at 25 Hz for 2m,  centrifuged, and stored at −80°C until processing.

RNA samples were thawed, 200 µL of additional Trizol was added, and tissue was further homogenized at 25 Hz for 2 m. RNA was extracted using the Direct-Zol RNA Miniprep kit (Zymo Research) following the manufacturer’s recommendations, but with an added wash step. On-column DNase treatment was not performed. The “rigorous” treatment protocol from the DNA-free kit (Ambion) was used to degrade DNA in RNA samples. Samples were confirmed DNA-free using PCR and gel electrophoresis for an arthropod-specific 28S rDNA (6, 37). The Qubit RNA HS Assay Kit (Invitrogen) was used to measure RNA concentration. Samples were diluted to 20 ng/µL, 2 µL of TATAA Universal RNA Spike I (tataabiocenter) was added to each sample, and 16 µL of the sample was converted to cDNA using SuperScript IV VILO Master Mix (Invitrogen).

### Relative-abundance and gene-transcript-level assays

Abundance and transcript-level assays were performed on testes nucleotide extracts. *Wolbachia* density was measured via qPCR as the relative abundance of the *Wolbachia* gene *ftsZ* and the single-copy gene *mid1*, which is conserved across the *Drosophila* genus (25, 77). *cifA-* and *cifB*-transcript levels were measured via RT-qPCR as the relative abundance of *cif* target to a spike-in control (described below). Since *cifs* are highly diverse, eight *cifA* primer sets and ten *cifB* primer sets were designed to capture the transcript levels of all variants encoded by our 10 focal *Wolbachia. cif* primers were designed using Primer3 in Geneious Prime (73) (Supplementary Table S7). All primers were efficient for relevant hosts and *Wolbachia*, except Type 3 *w*Tsa primers, which were not adequately tested due to insufficient transcript levels.

In RT-qPCR, Cq values are commonly normalized to an endogenously expressed gene to control for variation in RNA quality, reverse transcript levels, amplification, and pipetting. Endogenous controls must be consistently expressed across treatment groups to be valid. A single gene is unlikely to meet these criteria since we analyze transcript levels across nine *Drosophila* species. As such, we opted to normalize our qPCR results against an RNA spike-in control added to each sample after RNA dilution and prior to reverse transcript levels (described above). The spike-in control accounts for variation in reverse transcript levels, amplification, and pipetting that occurs after the spike-in is added to the sample and is considered equivalent or better than endogenous controls in some cases (78–80).

Samples were tested in triplicate using Powerup SYBR Green Master Mix (Applied Biosystems). Abundance FC was calculated as 2^–∆∆Ct^ of *ftsZ* and *mid1* relative to a random *w*Mel sample. Transcript levels FC was calculated as 2^–∆∆Ct^ of the *cif* target relative to the spike-in control compared to the average transcript levels of four reference samples with four unique *cif* copies: *cif_wMel[T1]_, cif_wRi[T2]_, cif_wTei [T4]_*, and *cif_wTri[T5]_*. FDR-corrected pairwise t tests were used to determine if *Wolbachia* density differed between systems. Analyses excluded samples with a Cq standard deviation exceeding 0.4 between triplicate measures.

### Development assays

We measured the developmental timing of fly species used in our study, in parallel. Stock bottles were cleared of flies, and all flies were collected after three days and held in a vial with fresh food for 24 h. Ten non-virgin *Wolbachia*-infected females were moved to a vial with a spoon with food, as described above for hatch-rate assays. Vials were monitored to determine the number of eggs laid and hatched every 3 h between 8 AM and 8 PM. After 20 to 30 eggs were laid, females were removed from vials, and the spoon was transferred to a vial containing 10 mL of fresh food. After eggs hatched, pupation was recorded every 6 h and adult emergence were recorded once daily. A vial was monitored until all adults emerged or there was no emergence for three days. Larval, pupal, and egg-to-adult development times were calculated as the time between the first egg hatch and the last larva pupation, the first pupation and the last adult emergence, and the first egg lay and last adult emergence per vial, respectively.

### Correlation analyses

We tested whether *Wolbachia* density, *cif*-transcript levels, or development time correlated with CI strength. Each *Wolbachia*–host pair represented a single sample in each analysis. PGLS regressions were used to test for relationships between BCa means of CI strength and the means of the traits above (density and transcript levels data were log2 transformed before taking the average) using caper in R (81). Analyses were performed with and without maximum-likelihood correction of *λ* and Akaike information criterion values were generated for each model. In all cases, correcting for *λ* yielded a preferred model, which we have reported.

### Phylogenetic signal

We investigated whether any of these traits exhibit phylogenetic signals using our *Wolbachia* phylogeny and data for CI strength, *Wolbachia* density, and *cif*-transcript levels. For *cif*-transcript levels, only Type 1 *cifs* were included in analyses, and only a single allele was selected from strains with multiple Type 1 copies (*cif_wHa[T1-1]_* and *cif_wBai[T1-1]_*). Two methods were used to investigate phylogenetic signals.

First, we calculated Fritz and Purvis’ *D* statistic using the *caper* R package, which estimates phylogenetic signal based on binary traits (55, 81). Binary traits were assigned as follows. *Wolbachia* density was low for *w*Ano, *w*Bai, *w*Boc, and *w*Tsa and high for other strains. *cifA*-transcript levels were high for *w*Ano, *w*Ri, and *w*Tri and low for other strains. *cifB*-transcript levels were high for *w*Ano, *w*Aur, *w*Ha, *w*Ri, *w*Tei, and *w*Tri and low for other strains. CI strength was treated as binary by either comparing strong CI (*w*Ano, *w*Aur, *w*Bai, *w*Ha, *w*Ri, *w*Tri) vs. all other strains or CI (strong-CI strains plus *w*Mel and *w*Boc) vs. non-CI strains. The *D* statistic compares the observed *D* value to alternative *D* values generated with simulated data based on a random phylogenetic pattern and Brownian motion using 1,000 permutations each. To investigate if larger phylogenies improve *D* statistic estimation, we used the Geiger package in R (82) to simulate 100 permutations of the *D* statistic for trees with 10, 25, 50, and 100 taxa (*λ* = 1 or 0) given the prevalence of our binary statistics.

Next, we calculated Pagel’s lambda (*λ*), using the phytools package in R, which estimates phylogenetic signal based on continuous traits (56, 83). A likelihood ratio test was used to compare our fitted value of *λ* to a model assuming no phylogenetic signal. Pagel’s *λ* was not calculated for CI since CI data is proportional. We used the mean of log2-transformed data for relative abundance and transcript levels.

### Figure generation

Figures were created using GGPlot2, and figure aesthetics were edited in Affinity Designer 1.8 (Serif Europe, Nottingham, UK).

## Supplementary Material

pgac099_Supplemental_Files

## Data Availability

All data are made publicly available in the supplement of this manuscript.
