# Supplementary material for: cifB-transcript levels largely explain cytoplasmic incompatibility variation across divergent Wolbachia
Source: PNAS Nexus. 2022 Jun 28;1(3):pgac099. doi: 10.1093/pnasnexus/pgac099 (PMC9364212; doi:10.1093/pnasnexus/pgac099)
Supplement: pgac099_Supplemental_Files [file pgac099_supplemental_files.zip › PNASNEXUS-PNASNEXUS-2022-00506-T-s01.docx]

**Supporting Information**


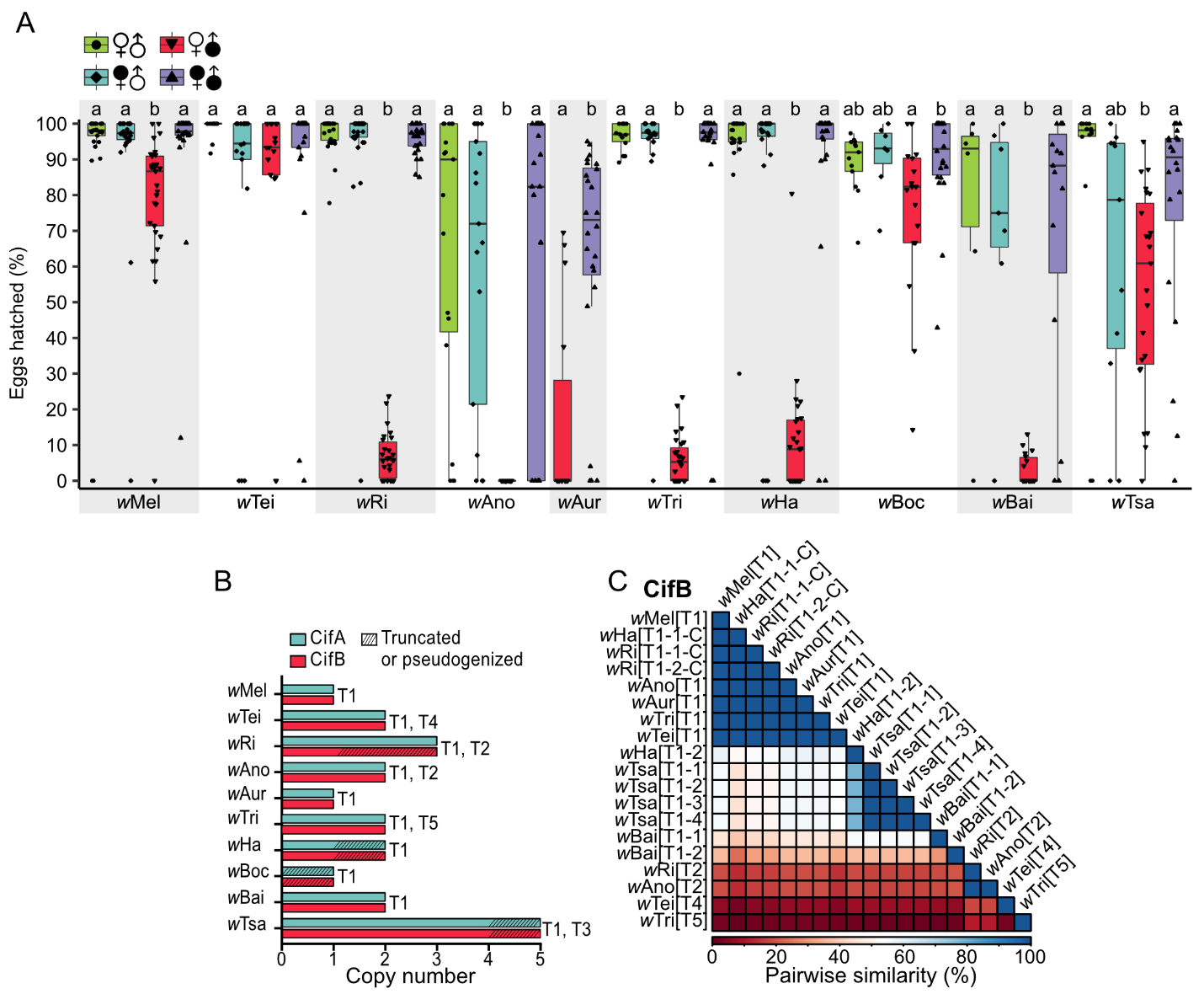


**Figure S1. CI-strength and Cif variation across *Drosophila*-associated *Wolbachia*. (A)** Hatch-rate assays display the percent of eggs hatched from mating pairs with different infection states. Significant differences are based on Kruskal-Wallis and Dunn’s tests between crosses for each strain. Significant differences are *P* < 0.05 = *, *P* < 0.01 = **, *P* < 0.001 = ***, and *P* < 0.0001 = ****. Exact *P*-values are reported in **Table S1**. **(B)** Cif copy number variation among our ten focal *Wolbachia*. **(C)** Similarity matrix displaying pairwise amino acid similarity for CifB proteins.


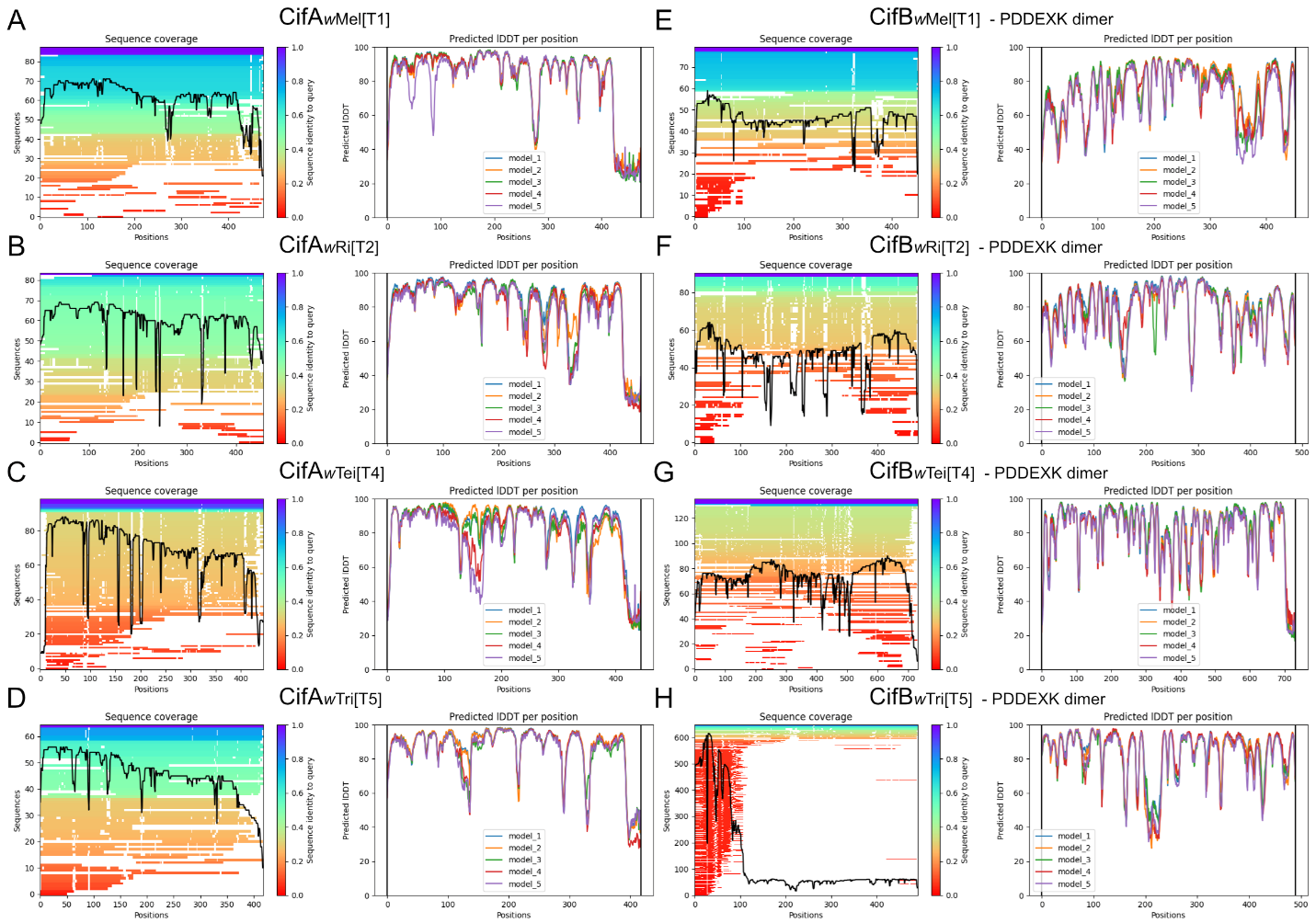


**Figure S2. Multiple sequence alignment results and Alphafold confidence for CifA and CifB PD-(D/E)XK** **pairs. (A-H, left)** Summary of the results from a multiple sequence alignment (MSA) generated by MMSeqs2 in the ColabFold pipeline. The black line indicates the number of sequences in the MSA that cover that position in the protein. Colored lines represent hits in the MSA where the color indicates sequence identity to the query. Sequences are organized by sequence coverage to the query, where higher coverage hits are at the top. **(A-H, right)** Alphafold structural confidence per amino acid position in the protein. Confidence is displayed as lDDT where higher values represent high confidence. Five models were produced for each protein. All five models are displayed here and are numbered in order of confidence. The model with the highest confidence for each protein (model 1) was used for all other analyses.


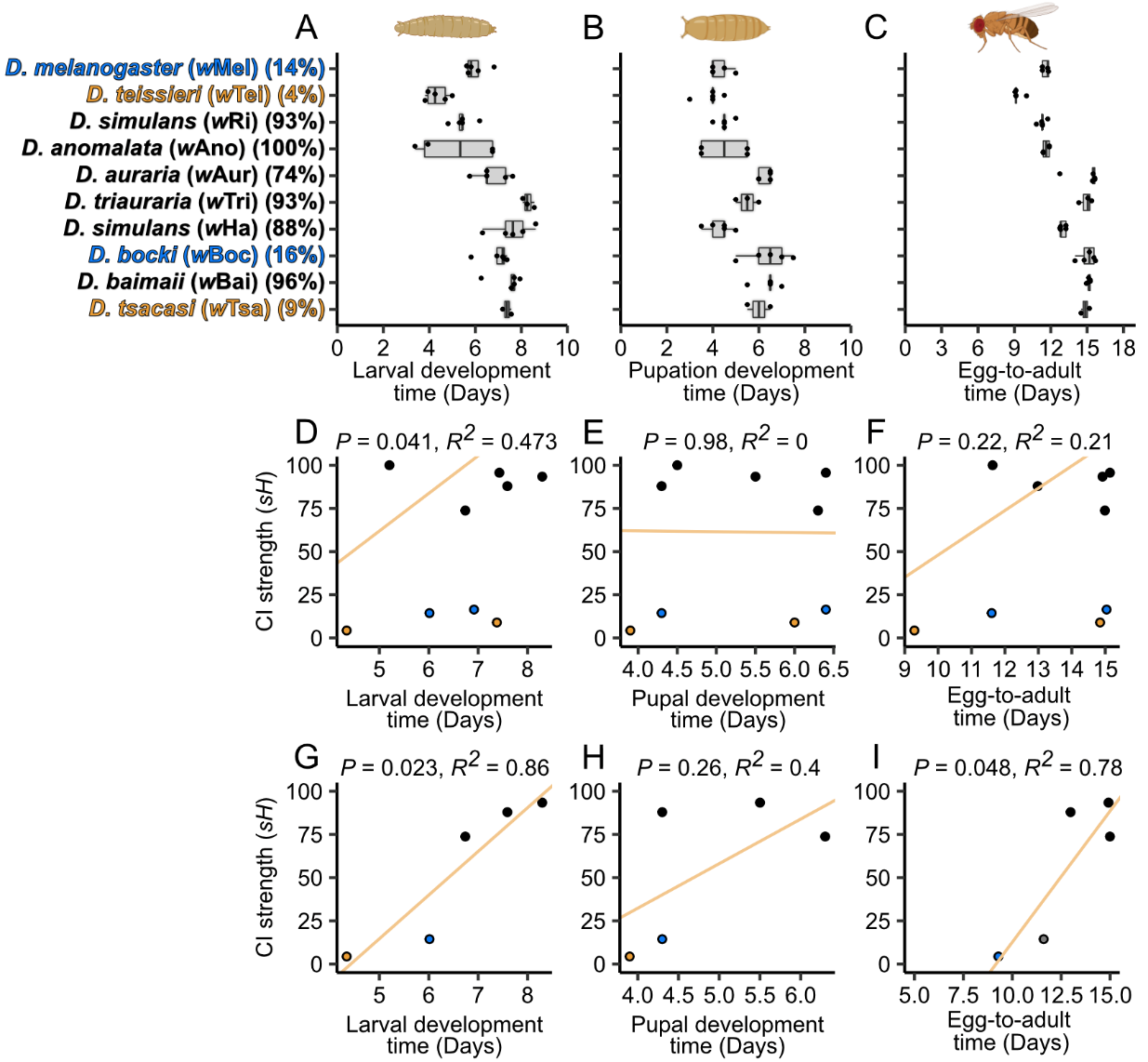


**Figure S3. Developmental timing and correlation with CI strength.** (A) The time between egg hatch and pupation. (B) The time between pupation and adult eclosion. (C) The time between egg lay and adult eclosion. Mean BCa estimates of CI strength are displayed in parentheses for reference. Names of strains are displayed in orange text if they do not cause significant CI, blue text if they cause weak CI, and black text if they cause strong CI. (**D-I**) Scatterplots showing the relationship between CI strength and development larval, pupal, or egg-to-adult development times. (**D-F**) Scatterplots include all strains while (**G-I**) only includes strains that did not have low *Wolbachia* densities. *P*-values, R^2^ values, and regression lines were calculated using a PGLS.


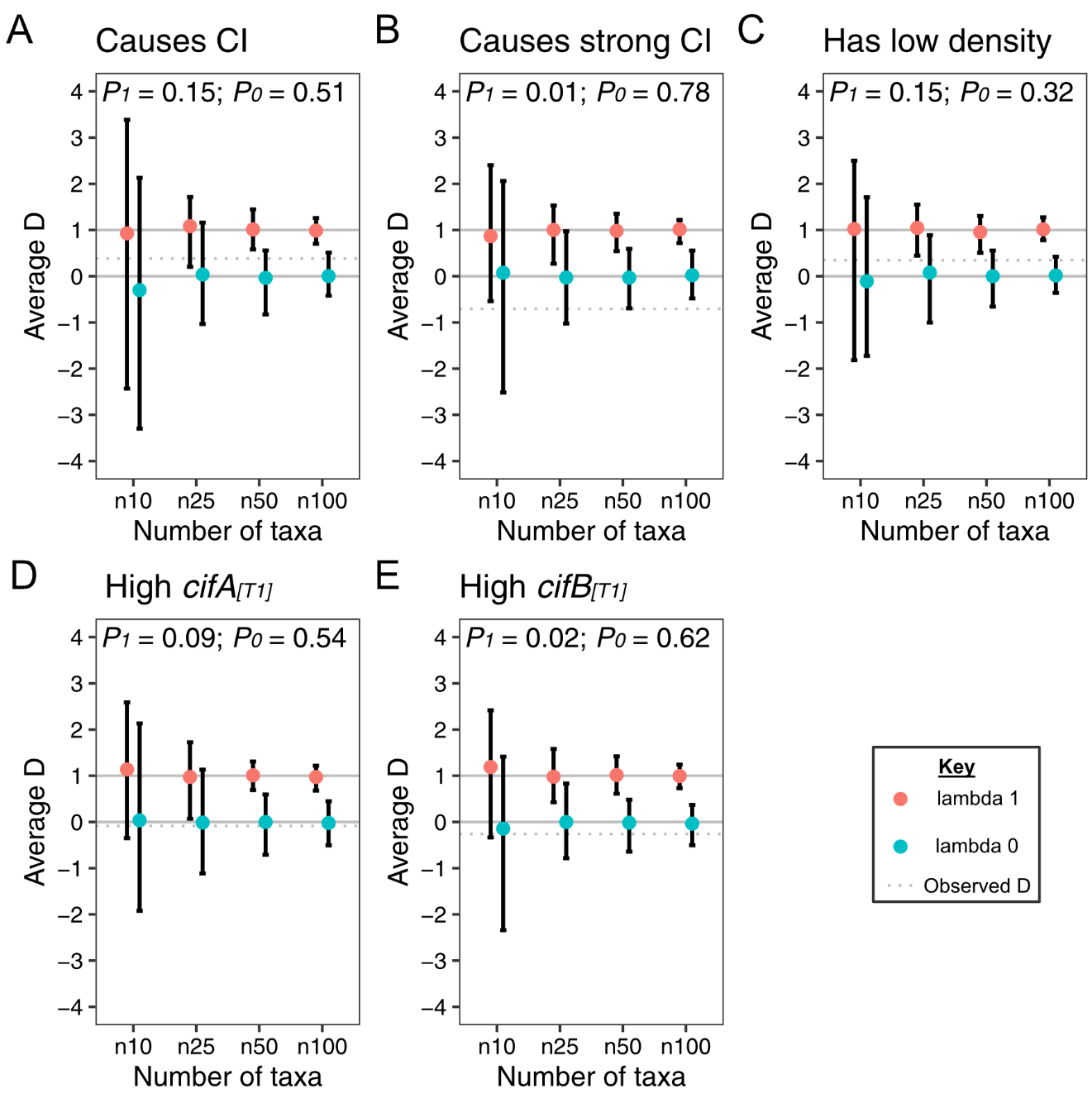


**Figure S4. Phylogenetic signals of binary traits using Fritz and Purvis’ *D* statistic.** The observed *D* statistic for each analysis is displayed with a dotted line. 95% confidence intervals are shown for 10, 25, 50, and 100 taxa based on Geiger simulations with 100 permutations each. Lambda of 1 indicates random placement relative to phylogeny; *P_1_* is the probability that *D* is similar to 1. Lambda of 0 indicates a phylogenetic signal consistent with a Brownian motion model of evolution; *P_0_* is the probability that *D* is similar to 0. Analyses are displayed for whether strains **(A)** cause CI, **(B)** have low-*Wolbachia* density, **(C)** express high levels of *cifA_[T1]_*, **(D)** cause strong CI, or **(E)** express high levels of *cifB_[T1]_*.

**Table S1. Exact *P*-values from this study’s statistical analyses.**

**Table S2. HHpred results for CifA and CifB proteins.** Only hits with greater than 80% probability were recorded, and only the top hit was recorded for annotations that overlap a portion of the protein.

**Table S3. Summary of Alphafold structural confidence and pairwise structural similarities.**

**Table S4. Analysis of CI-strength covariates using phylogenetic generalized least squares regression to account for *Wolbachia* phylogenetic relationships.**

**Table S5. Analysis of CI-strength covariates using phylogenetic generalized least squares regression to account for host phylogenetic relationships.**

**Table S6. Lines used in this study and their associated genomes. Uninfected lines were derived from each of these using tetracycline and the + was replaced with - in each study name.**

**Table S7. Primers, targets, and PCR conditions used in this study.**
